# Lung Disease in Sjögren’s Syndrome: Insights From a Reference Center’s Population

**DOI:** 10.7759/cureus.73734

**Published:** 2024-11-15

**Authors:** Catarina Silva Araújo, Francisco Laranjeira, Mariana Simão de Magalhães, Ana Oliveira, Martinha M Vale, Inês Furtado, Fátima Farinha, Mariana Brandão, Ivone Valadão

**Affiliations:** 1 Internal Medicine, Unidade Local de Saúde de Braga, Braga, PRT; 2 Internal Medicine, Hospital da Luz Lisboa, Lisboa, PRT; 3 Internal Medicine, Unidade de Caldas da Rainha, Unidade Local de Saúde do Oeste, Caldas da Rainha, PRT; 4 Internal Medicine, Unidade Local de Saúde de Santo Antônio, Porto, PRT; 5 Autoimmunity and Neurosciences Group, Unit for Multidisciplinary Research in Biomedicine, Institute of Biomedical Sciences, University of Porto (UMIB, ICBAS-UP), Porto, PRT; 6 Clinical Immunology Unit, Unidade Local de Saúde de Santo Antônio, Porto, PRT

**Keywords:** intersticial lung disease, lymphocytic interstitial pneumonia, nonspecific interstitial pneumonitis, primary sjögren's syndrome

## Abstract

Background

Sjögren's syndrome (SS) is a chronic systemic autoimmune disorder characterized by lymphocytic infiltration of the exocrine glands. Although primarily affecting these glands, the syndrome can also involve several organs. Interstitial lung disease is one of the most severe complications associated with SS.

Objectives

This study aims to analyze the characteristics of lung involvement in patients with primary SS from a reference medical center's population.

Methods

Retrospective, longitudinal study of primary SS patients diagnosed until December 2022. Clinical and laboratory data were collected and subjected to statistical analysis using SPSS®. A 95% confidence interval was considered, with statistical significance set at p<0.05.

Results

A total of 126 patients were included, 95.2% of whom were females, with a mean age at diagnosis of 50 years. The median EULAR Sjögren's Syndrome Disease Activity Index (ESSDAI) at diagnosis was 3, which decreased to 1 at the last evaluation. Based on the ESSDAI lung domain, nine patients presented with lung disease at diagnosis. This number increased to 18 by the last assessment. Thoracic tomography showed lung disease in 30 patients, with bronchiectasis being the most common finding. Ten patients presented with interstitial lung disease. Pulmonary function tests were abnormal in 18 patients, and the diffusing capacity of the lungs for carbon monoxide was reduced in 32. Patients with lung disease were more likely to experience a dry cough (p<0.01), have positive SSA antibodies (p<0.05), antinuclear antibody (ANA) >1/320 (p<0.01), positive rheumatoid factor (p<0.01), and low C4-complement during follow-up (p<0.05).

Conclusions

The authors found an association between clinical and analytical findings and the presence of lung disease in SS patients. Further research is essential to identify optimal predictors of SS-associated lung disease, facilitating early diagnosis and intervention for both SS and its related lung complications.

## Introduction

Sjögren's syndrome (SS) is a chronic systemic autoimmune disorder characterized by lymphocytic infiltration of the exocrine glands [1,2]. SS may occur alone, being classified as primary, or in association with another rheumatic disease, categorized as associated [1,2]. The most common symptoms are dry eyes and mouth, but it can affect multiple organs [1,2]. There is no cure for the disease, so the main objectives are to control symptoms, prevent complications, and maintain quality of life [1].

Interstitial lung disease is one of the most severe complications associated with SS, with a reported prevalence of 20% [2,3]. It can present various patterns: lymphocytic interstitial pneumonia (the most typical but less common pattern), nonspecific interstitial pneumonia (the most common), usual interstitial pneumonia, and organizing pneumonia [3,4]. Pulmonary involvement in SS can have diverse clinical presentations and symptoms that overlap with other pulmonary diseases, such as a dry cough, making diagnosis challenging [4]. Early recognition and appropriate treatment of pulmonary involvement are crucial because treatment strategies are evolving and becoming more effective.

This study aims to analyze a population of patients with SS to determine if there are markers that could be used as predictors of organ involvement associated with the presentation of lung disease.

## Materials and methods

Study design and population

This retrospective and longitudinal study included all the patients diagnosed with SS according to the ACR/EULAR 2016 "Classification Criteria for primary Sjögren's Syndrome" [5], followed at our center from 1987 until December 2022. Lung involvement was defined according to the EULAR Sjögren's syndrome disease activity index (ESSDAI) score [6] pulmonary domain: at least 1 point at the last evaluation, after excluding any other causes. Patients with chronic lung disease of uncertain cause were excluded.

Study measures

Clinical and laboratory data were collected from the patient’s clinical files: sex, date of birth, age at diagnosis, duration of symptoms until diagnosis, ESSDAI score at the diagnosis and at last follow-up, salivary gland biopsy, immunology blood test results, presence of dry cough, thoracic CT results, pulmonary function tests results, diffusing capacity of the lungs for carbon monoxide (DLCO), cause and time of death.

Ethics statement

All procedures were in accordance with the ethical standards of the institution and with the Declaration of Helsinki. Due to its retrospective nature, informed consent was not required. All reasonable measures were taken to protect the privacy and confidentiality of patient data. No new data were obtained specifically for the purpose of this study.

Statistical analysis

The collected data was analyzed using the software IBM SPSS® version 23.0. A 95% confidence interval was considered, with statistical significance for p<0.05. The Kolmogorov-Smirnov test was used to test the existence of a normal distribution in continuous quantitative variables. The mean and standard deviation are presented for continuous variables with a normal distribution. The median and interquartile range are presented for those with a non-normal distribution. Categorical variables are presented as absolute numbers and percentages. Between-group analyses in categorical variables were performed with the Chi-square and Fisher’s exact tests. For continuous variables, the Kruskal-Wallis and Man-Whitney U tests were used.

## Results

The sample included 126 patients, with a mean age at diagnosis of 50 years (Table 1). The median duration of symptoms at diagnosis was five years, and the median duration of follow-up was 11 years. During this period, 12 patients died. The median ESSDAI at diagnosis was 3 points and at the last evaluation was 1 point. The number of patients with pulmonary disease associated with SS increased from the moment of diagnosis to the last evaluation (11 vs 18). The immunology blood test results are described in Table 1.

**Table 1:** Sample characterization. *Mean ± standard deviation; **Median (interquartile range); *** Reference values in Appendix 1; ◊: At least once during follow-up; ANA: Antinuclear antibodies; Anti-SSA: Anti-Sjögren's syndrome-related antigen A; Anti-SSB: Anti–Sjögren's syndrome-related antigen B; ESR: Erythrocyte sedimentation rate; ESSDAI: EULAR Sjögren's syndrome disease activity index; NS: Non significant.

Variable		Full Sample (n=126)
Gender	Male	6 (4.8%)
	Female	120 (95.2%)
Age at diagnosis (years)*		50.2±15.4
Time to diagnosis (years)**		5 (2-9)
Time to diagnosis (group of years)	0-4	92 (49.2%)
	5-9	37 (29.4%)
	≥10	27 (21.4%)
Follow-up duration (years)**		11 (7-13)
Death		12 (9.5%)
Positive minor salivary gland biopsy (n=98)		87 (88.8%)
ESSDAI at diagnosis (n=97)**		3 (1.5–4)
ESSDAI Pulmonary domain activity level at diagnosis	0 = No	88 (90.7%)
	1 = Low	6 (6.2%)
	2 = Moderate	3 (3.1%)
	3 = High	0 (0%)
Last ESSDAI evaluation**		1 (0-2)
ESSDAI Pulmonary domain activity level at last evaluation	0 = No	108 (85.7%)
	1 = Low	11 (8.7%)
	2 = Moderate	5 (4%)
	3 = High	2 (1.6%)
Immunology Blood Tests***	Positive anti-SSA	105 (83.3%)
	Positive anti-SSB	62 (88.1%)
	Positive anti-alpha-fodrin (n=88)	26 (29.5%)
	Positive Rheumatoid Factor	69 (54.8%)
	ANA ≥ 1/160 during follow-up	111 (88.1%)
	ANA ≥ 1/320 during follow-up	98 (77.8%)
	Presence of cryoglobulins ◊ (n=106)	17 (16%)
	Low C3 at diagnosis	13 (10.3%)
	Low C3 during follow-up	22 (17.5%)
	Low C4 at diagnosis	13 (10.3%)
	Low C4 during follow-up	24 (19%)
	ESR at diagnosis (mm/h)**	29.5 (16.8-57)
	Highest ESR during follow-up (mm/h)*	63.3 ± 27.5
	IgG at diagnosis (mg/dL)**	1500 (1205-2180)
	Highest IgG during follow-up (mg/dL)**	1740 (1332-2496.5)

A comparison between the patients with SS with and without associated lung disease is made through Tables 2-3. Blood test reference values are presented in Appendix 1.

**Table 2:** Comparison between patients with Sjögren’s syndrome presenting with and without associated lung disease. 1: One or more points on ESSDAI’s pulmonary domain; 2: At least once during follow-up; *mean ± SD; **median (interquartile range); *** reference values in appendices; ◊: At least once during follow-up; ANA: Antinuclear antibodies; Anti-SSA: Anti–Sjögren's syndrome-related antigen A; Anti-SSB: Anti–Sjögren's syndrome-related antigen B; ESR: Erythrocyte sedimentation rate; ESSDAI: EULAR Sjögren's syndrome disease activity index; NS: Non-significant; U: Mann-Whitney U test statistic; χ²: Chi-square test statistic; Z Standardized value associated with the Mann-Whitney U test.

Variable		Lung Disease in Sjögren’s Syndrome Patients^1^				
		No (n=108)	Yes (n=18)	x^2^	U / Z	p
Gender	Male	5 (4.6%)	1 (5.6%)	0.029	-	NS
	Female	103 (95.43%)	17 (94.4%)			
Age at diagnosis (years)*		50.3±15.4	50.1±15.6	-	952 / -0.139	NS
Time to diagnosis (years)**		5 (2-9)	3.5 (2-6)	-	951.5 / -0.073	NS
Death		10 (9.3%)	2 (11.1%)	0.061	-	NS
ESSDAI at diagnosis (n=97)**		3 (1-4)	3 (2-4)	-	482,5 / -1.022	NS
Positive minor salivary gland biopsy (n=98)		76 (89.4%)	11 (84.6%)	0.260		NS
Immunology Blood Tests	Positive Anti-SSA	89 (82.4%)	16 (88.9%)	0.467	-	NS
	Positive Anti-SSB	53 (49.1%)	9 (50%)	0.005	-	NS
	Positive Anti-alpha-fodrin (n=88)	22 (30.6%)	4 (25%)	0.194	-	NS
	Positive Rheumatoid Factor	55 (55.6%)	14 (77.8%)	6.920	-	<0.05
	ANA ≥ 1/160 during follow-up	95 (88%)	16 (88.9%)	0.013	-	NS
	ANA ≥ 1/320 during follow-up	82 (75.9%)	16 (88.9%)	1.500	-	NS
	Presence of Cryoglobulins ◊ (n=106)	15 (16.7%)	2 (12.5%)	0.175	-	NS
	Low C3 at diagnosis	10 (9.3%)	3 (16.7%)	0.915	-	NS
	Low C3 during follow-up	18 (16.7%)	4 (22.2%)	0.330	-	NS
	Low C4 at diagnosis	11 (10.2%)	2 (11.1%)	0.014	-	NS
	Low C4 during follow-up	17 (15.7%)	7 (38.9%)	5.362	-	<0.05
	ESR at diagnosis (mm/h)	37.1 ±26.8	36±25.9	-	961.500 / -0.0.73	NS
	Highest ESR during follow-up (mm/h)	62.6 ±27.3	65.4±29.7	-	932 / -0.279	NS
	IgG at diagnosis (mg/dL)	1845.5±956.3	1693.3±775.3	-	914 / -0.345	NS
	Highest IgG during follow-up (mg/dL)	2030±994.9	2079±886.7	-	873.5 / -0.629	NS

**Table 3:** Comparison between patients with Sjögren’s syndrome presenting with and without associated lung disease, concerning clinical manifestations, thoracic tomography, respiratory function tests, and diffusing capacity of the lungs for carbon monoxide. 1: One or more points on ESSDAI’s pulmonary domain; * DLCO <78%; DLCO: Diffusing capacity of the lungs for carbon monoxide; NS: Non-significant; χ²: Chi-square test statistic.

Variable			Lung Disease in Sjögren’s Syndrome Patients^1^			
		No (n=108)		Yes (n=18)	x^2^	p
Dry cough		15 (13.9%)		11 (61.1%)	21.008	<0.001
Thoracic tomography (n=86)	Abnormal	40 (58.8%), n=68		17 (94.4%)	8.080	<0.01
	Bronchiectasis	15 (22.1%)		10 (55.6%)	7.745	<0.01
	Interstitial Pneumonia	1 (1.5%)		9 (50%)	32.619	<0.001
	Ground Glass	7 (10.3%)		8 (44.4%)	11.527	<0.01
	Fibrosis	6 (8.8%)		7 (38.9%)	10.026	<0.01
Interstitial pattern (n=10)	Nonspecific Interstitial Pneumonitis	0 (0%)		5 (55.6%); n=9	38.058	<0.001
	Lymphocytic interstitial pneumonia	0 (0%)		4 (44.4%); n=9		
	Hypersensitivity Pneumonitis	1 (100%); n=1		0 (0%)		
Pulmonary Function Tests (n=66)	Normal	54 (81.8%)		12 (66.7%)	8.660	<0.05
	Obstructive	10 (15.2%)		2 (11.1%)		
	Restrictive	2 (3%)		3 (16.7%)		
	Mixed defects	0 (0%)		1 (5.6%)		
Reduced DLCO* (n=78)		23 (37.1%), n=62		9 (56.3%), n=16	1.928	NS

Table 2 establishes general comparisons with particular emphasis on immunological studies. There we can see that the Sjögren's patients presenting with lung disease showed a more frequent positive rheumatoid factor (RF) and C4 consumption during follow-up (p<0.05) compared to those without pulmonary involvement.

Table 3 focuses on the pulmonary investigations undertaken by all Sjögren's patients, including clinical manifestations, lung CT, respiratory function tests, and diffusing capacity of the lungs for carbon monoxide (DLCO). It shows the different patterns of changes identified in each group of patients, those who had lung disease and those who did not. As expected, lung disease patients presented significantly more dry cough and abnormalities in thoracic CT and pulmonary function tests. Concerning CT scans, bronchiectasis was the most common finding, followed by interstitial lung patterns. Although no significant difference in DLCO analysis between groups was found, the data showed that this marker was lower in those with lung involvement.

Mortality analysis was not performed because only one death was directly associated with Sjögren’s lung disease (Table 4).

**Table 4:** Causes of death.

Cause of death	(n=12)
Malignant neoplasm	3 (25%)
Infection	2 (16.6%)
Sjogren’s associated lung disease	1 (8.3%)
Heatstroke	1 (8.3%)
Liver failure	1 (8.3%)
Unknown	4 (33.3%)

## Discussion

Although in many cases, SS symptoms go unnoticed, during the evolution of the disease, they can dramatically impact patients' quality of life [7]. The median duration of symptoms before diagnosis emphasizes the need for clinicians to be more aware of this syndrome to ensure timely diagnosis. Patients with severe presentations usually receive an earlier diagnosis, while those with milder symptoms are frequently overlooked. However, even these patients can develop severe complications over time and should not be neglected [7].

Regarding the ESSDAI, the improvement in the score observed at the last visit compared with the diagnosis (median of 1 vs. 3 points) may result from the treatments instituted after diagnosis.

The literature estimates pulmonary involvement in SS to be between 10% and 20% [4,8]; this aligns with our findings of 14.3% lung involvement. From the first to the last evaluation, the number of patients diagnosed with pulmonary disease increased (9.3% vs. 14.3%), demonstrating that despite follow-up at a reference center with standard care therapy, current medical treatments remain insufficient to fully prevent or cure lung disease.

This study aimed to identify clinical variables that could help detect patients at higher risk of lung disease among those with SS (Tables 1-2).

Although positive anti-SSA and anti-SSB antibodies have been described as risk factors for SS lung disease [2,9], there was no significant difference between groups in our sample. Perhaps a larger sample size might reveal significant results.

The presence of a positive RF has been associated with more severe presentations of SS, including lung disease [2,9,10]. This study also found a higher prevalence of positive RF in those with lung disease, suggesting that patients with positive RF should be monitored more closely. Those with negative RF might benefit from repeating this test during follow-up for lung disease risk reassessment.

In our full sample, the percentage of patients with antibodies against alpha-fodrin was similar to those reported by other authors [11,12]. This biomarker failed to show any association with lung disease, which is consistent with other studies that were also unable to establish this link [12,13].

The number of patients with low C3 and C4 levels (Table 1) was very similar to those reported in the literature (1%-15% at diagnosis and around 20% during the course of the disease) [14,15-17]. Patients with lung disease more frequently exhibited low C3 and C4 levels both at diagnosis and during follow-up. Despite this tendency, a statistically significant difference was observed only for low C4 levels during follow-up. Other studies have shown a correlation between low C3 and C4 levels and more severe forms of the disease, with extraglandular manifestations (including lung disease) [15,18,19].

A high IgG level is usually concerning due to its association with an increased risk of lymphoma and elevated disease activity [10]. However, in this study, no correlation was found with pulmonary involvement. Other studies have similarly failed to demonstrate this relationship [20].

The erythrocyte sedimentation rate was expected to be higher in those with lung disease [20], but no significant difference was found between groups in this population.

Although many blood immunologic tests were analyzed, only RF positivity and C4 consumption showed significant differences, with both being more frequent in those with lung disease. Further studies are needed to better understand the associated risks of each predictor.

Dry cough was more frequently observed in patients with lung disease but was not exclusive to this group (Table 3). Although nonspecific, the presence of this symptom should prompt physicians to consider the progression to pulmonary involvement and to request pulmonary function tests and thoracic imaging earlier in the disease course [8]. Closely monitoring these patients' symptoms can lead to earlier diagnosis and treatment.

Lung evaluations revealed multiple patterns of lung disease, with bronchiectasis and interstitial lung disease being the most frequent. Other authors have described all the CT findings presented in SS [4]. Even though lymphocytic interstitial pneumonia is the most typical pattern in SS-associated lung disease, it was not the most frequent in our study. Similar findings have been reported by other studies, which also indicated that this typical pattern is less common [3,4]. The small sample size is a significant limitation, preventing a more detailed analysis of CT findings.

Regarding mortality, only one patient died due to SS lung disease. While this is a positive finding, it prevents a clear and comprehensive analysis of prognosis markers. More research is needed on this topic, along with the development of targeted therapies that can reverse SS lung disease.

This study is limited by its retrospective design and the relatively small sample size. Patients may have other lung conditions (such as chronic obstructive pulmonary disease), making it challenging to attribute alterations in pulmonary function tests solely to SS. Prospective and multicenter studies are necessary to expand our understanding of this disease.

## Conclusions

Despite the high morbidity associated with pulmonary manifestations, the mortality analysis revealed a relatively low rate of deaths attributable to SS-associated lung disease in this sample.

Dry cough, positive RF, and low C4 levels are associated with SS-associated lung disease, making them potential biomarkers for identifying at-risk patients. Although serologic biomarkers cannot yet be reliably used to evaluate pulmonary involvement, this study suggests that some may increase clinicians' suspicion. Further research on prognostic factors, target-organ disease biomarkers, and specific interventions in this patient population is needed.

## Appendices

Appendix 1 

**Table 5:** Reference values for laboratory tests. SS: Sjögren's syndrome.

Laboratory tests	Reference Values
Anti-SSA (U/mL)	<10
Anti-SSB (U/mL)	<10
Anti-alpha-fodrin (U/mL)	<18
Rheumatoid Factor (U/mL)	<20
Cryoglobulins (µg/mL)	<20
C3 (mg/dL)	81-167
C4 (mg/dL)	11-24
Erythrocyte Sedimentation Rate (mm/h)	0-20
IgG (mg/dL)	29-226
